# Enhancing supply chain management in the physical internet: a hybrid SAGA approach

**DOI:** 10.1038/s41598-023-48384-y

**Published:** 2023-12-06

**Authors:** Weiqi Yan, Nan Li, Xin Zhang

**Affiliations:** 1 Xi’an University of Finance and Economics, Xi’an, 710049 China; 2 Xi’an Polytechnic University, Xi’an, 710055 China

**Keywords:** Engineering, Mathematics and computing

## Abstract

This paper introduces an advanced inventory replenishment optimization approach tailored for the Physical Internet (PI), addressing the dynamic and complex nature of this environment. We propose a hybrid Simulated Annealing–Genetic Algorithm (SA–GA), engineered to optimize the balance between exploration and exploitation, ensuring adaptability and efficiency in a variety of PI contexts. The study also presents an enriched mathematical model integrating dynamic demand, and multi-objective optimization. The SA–GA algorithm emerges as a novel contribution, characterized by its computational efficiency and adaptability, marking an advancement in PI inventory management. The incorporation of real-time data analytics in our dynamic inventory replenishment strategy enhances adaptability and responsiveness, while the robust mathematical model offers a versatile tool for both theoretical analysis and practical application. Collectively, these innovations help bridge existing gaps in PI inventory management and serve as a reference for other similar studies.

## Introduction

The Physical Internet (PI) is a groundbreaking concept that transforms traditional logistics and supply chain management by integrating advanced information technologies, standardized processes, and modular containers to create a highly efficient, sustainable, and responsive global logistics system. Emerging from the collaborative efforts of researchers, industry professionals, and policymakers, PI aims to address the escalating challenges of complexity, inefficiency, and environmental impact inherent in traditional supply chains^1^. Modeled after the digital internet, PI leverages an interconnected and efficient physical network, comprising standardized modular containers, intelligent logistics nodes, and collaborative resource and information sharing^2^. This innovative concept holds great promise in enhancing supply chain sustainability and cost reduction^3^.

In the pursuit of realizing the full potential of PI, optimizing inventory replenishment becomes a paramount endeavor^4^. Inventory replenishment pertains to the process of replenishing inventory levels to meet the demand of downstream entities, such as retailers^4^. In traditional logistics networks, inventory replenishment typically adheres to predetermined strategies, wherein the source of goods, replenishment methods, and storage locations are fixed^5^.

Conventional inventory replenishment models and optimization techniques have long been studied and applied in supply chain management. These models seek to strike an optimal balance between inventory holding costs and ordering costs while ensuring the fulfillment of downstream entities' requirements. Classical methods like economic order quantity (EOQ) and its variations have been widely employed to determine optimal order quantities and reorder points in static and non-interconnected supply chain networks.

However, the advent of PI has introduced a new dynamic to inventory replenishment. The incorporation of standardized modular containers, intelligent logistics nodes, and advanced information-sharing capabilities in the PI environment enables agile and adaptable decisions regarding inventory replenishment strategies. Through collaborative efforts among suppliers, PI centers, and retailers, resources are shared, leading to enhanced efficiency and cost-effectiveness in the replenishment process.Compared to traditional supply chains characterized by linear, static, and siloed operations, PI embodies dynamism, interoperability, and fluidity. Where traditional models grapple with inefficiencies, lack of visibility, and rigidity, PI offers real-time data exchange, modularization, and dynamic routing, heralding a new era of agile, efficient, and sustainable logistics and supply chain management.

Despite the growing enthusiasm surrounding the PI concept, the literature on inventory replenishment within the PI context remains relatively limited. Existing research predominantly concentrates on the design and optimization of PI-supported logistics networks, with a specific focus on transportation and route optimization^6,7^. Mohamed et al. Literature^8^ discusses the transportation problem of applying PI in the interconnected urban logistics environment, proposes a comprehensive modeling method for multi-time periods, multi-regions, heterogeneous fleets and multi-trips, and discusses the model solvability and algorithm performance. Dang and Kim^9^ first proposed a service-oriented information framework (SOA) for PI infrastructure, which can realize universal interconnection of various entities in the PI environment to provide effective logistics services. Lemens et al.^10^ studied the synchronous transportation mode in PI environment, which can greatly reduce carbon emissions and transportation costs. Salez et al. Literature^11^ explores the conversion of transportation modes between PI and hubs in a PI environment, and uses simulation methods to explore its practicality.Noteworthy investigations have also delved into the optimization of inventory under the PI environment. Scholars have also carried out meaningful explorations on the inventory optimization problem under the PI environment.Pan et al.^12^ contributed to the field of Physical Internet (PI) inventory management by proposing a source selection strategy for goods within the PI framework. Their research explored the latest directions related to PI inventory management, enhancing the understanding of efficient goods sourcing in interconnected PI networks.Yang Yanyan et al.^13,14^ conducted a comprehensive comparison of inventory replenishment strategies in traditional and PI-enabled logistics networks. They constructed a mixed integer nonlinear programming model and evaluated the efficiency of inventory control strategies using the simulated annealing algorithm. Their work shed light on the advantages of PI-based inventory management in logistics networks. Shoufeng et al.^15^ delved into the multi-scenario production-inventory-distribution problem within the PI environment. They proposed an integrated production-inventory-distribution model to optimize the performance of logistics networks. Their experiments demonstrated that adopting the PI approach can lead to significant cost savings in production-inventory-distribution processes. Nouiri et al.^16^ explored the inventory management challenges in logistics networks under both traditional and PI environments. They applied simulation methods to demonstrate the superiority of PI logistics networks, particularly under interruption conditions, highlighting the resilience and robustness of the PI approach in dealing with disruptions. Lingrong et al.^17^ discussed the collaborative replenishment problem of Sup-ply-hub under the condition of uncertain product demand and replenishment lead time. With the goal of minimizing supply chain operating costs, they established supply chains under three different replenishment strategies. Replenishment model. Jianghai et al.^18^ studied the dynamic inventory sharing problem of airline multi-base inventory system in a single cycle by designing a polynomial algorithm to solve the network flow model. Pan et al.^12^ proposed a supply selection strategy in the PI environment and determined the latest research directions related to PI inventory management.

Although the above studies have contributed to inventory optimization in the PI environment. Traditional inventory management methods, while effective in conventional supply chains, are often inadequate in the dynamic, intricate, and interconnected environment of the PI. Fluctuating demands, diverse supply sources, and the imperative for real-time decision-making have highlighted the need for more advanced, adaptive, and responsive inventory management strategies.In response to these challenges, the motivation behind our study stems from a pressing need to develop solutions that are tailored to the complexities inherent in the PI.Scholars have proven that multi-objective optimization problems can be effectively solved by using appropriate hybrid algorithms^19,20^.

Our focus is on optimizing inventory replenishment to achieve a balance of cost-effectiveness, efficiency, and agility in supply chain operations. By introducing the hybrid SAGA approach, we aim to bridge existing gaps in literature and practice, offering a methodology that is adept at navigating the multifaceted landscape of PI-based supply chain management. The SAGA approach is designed to deliver optimal solutions that are both theoretically robust and practically applicable in diverse PI scenarios. In this study, we propose two unique solutions to address this challenge. Strategy 1 is designed for the dynamic PI environment, focusing on minimizing various costs and efficiently moving goods between retailers and PI centers. Strategy 2, on the other hand, adheres to the traditional approach of replenishing stocks through suppliers whenever there is a shortage at the PI centers or retailers. Our proposed inventory optimization model considers key factors, including source selection strategies, inventory holding costs, ordering costs, transportation costs, and penalty costs. By utilizing this hybrid algorithm, we aim to optimize the replenishment process within a dynamic and interconnected PI network with the overall goal of minimizing the total cost while meeting the retailer's inventory requirements.

Through in-depth case studies, we carefully evaluate the algorithm's performance, yielding valuable insights into the significant benefits of optimizing inventory replenishment in a PI environment. Our research can not only provide a new solution to the inventory optimization problem in the PI environment but also provide new management enlightenment for decision-makers.

The main contributions of this paper are as follows:

- Development of a novel SA–GA hybrid optimization algorithm for inventory management in the Physical Internet (PI) environment.
- Comprehensive comparative analysis demonstrating the superior performance of the SA–GA algorithm against traditional approaches.
- Application of the SA–GA algorithm in a real-world case, showcasing practical benefits in cost minimization and supply chain efficiency.
- Enhanced algorithmic balance between exploration and exploitation, improving convergence speed and solution accuracy.

The remainder of this paper is structured as follows: Section "Questions raised" presents a detailed review of the relevant literature, laying the groundwork for our research. Section "Algorithm design" introduces the methodology, including the development of the SA–GA hybrid optimization algorithm and its application in inventory management within the Physical Internet (PI) environment. Section "Case verification" details the experimental setup and data used to validate our approach. In Section "Discussion", we discuss the results of our experiments, highlighting the effectiveness of the SA–GA algorithm compared to traditional methods. Section "Conclusion" offers a comprehensive discussion of these results, delving into the implications and potential applications of our findings. Finally, Section "Limitations and future work" concludes the paper with a summary of our contributions, limitations of the current study, and directions for future research.

## Questions raised

### Problem description

Within the complex framework of the Physical Internet (PI) ecosystem, the urgent problem of inventory replenishment emerges to dynamically determine the optimal point of purchase, order size and reorder point to suit the needs of retailers. This intricate problem requires optimizing the replenishment process while judiciously considering multiple factors including inventory holding costs, ordering costs, shipping costs, and penalty costs. The overall goal is to minimize cumulative costs while meeting the retailer's specific inventory requirements. In this study, we propose two unique solutions specifically designed for the inventory replenishment problem.

**Figure 1:**
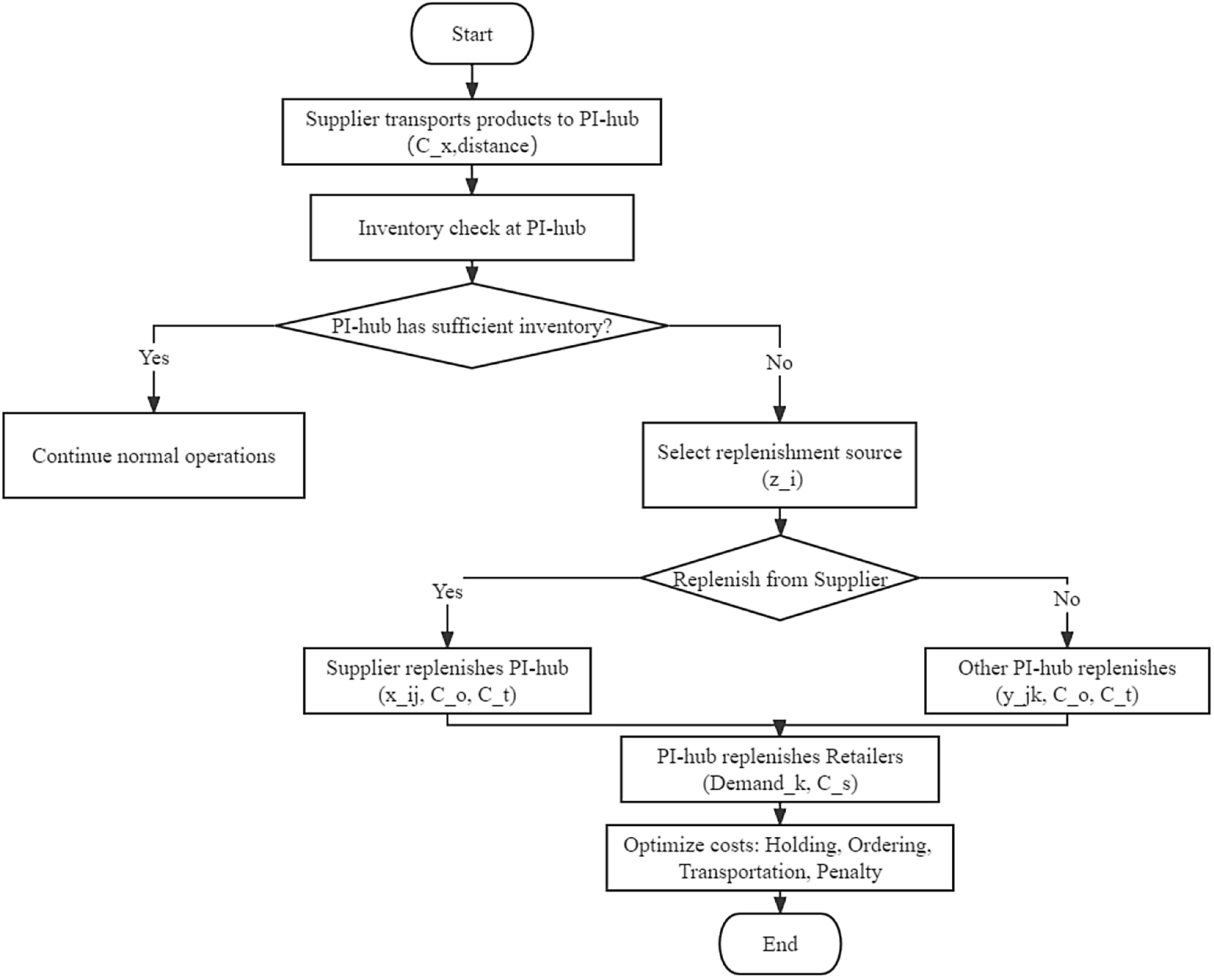
Inventory replenishment process in PI environment.

Strategy 1 is tailored for dynamic replenishment in a PI environment, as shown in Fig. 1. This strategy is based on minimizing the costs associated with stock holding, ordering, shipping, and fines. More precisely, the movement of goods mainly takes place between retailers and PI centers, thus fulfilling the demand for goods. However, if the existing inventory in all PI Centers is insufficient to meet the demand, replenishment must be done through the supplier.

On the other hand, Strategy 2 represents the traditional replenishment approach, where either the PI center or retailer is out of stock and needs to be replenished through suppliers.

### Assumptions

1. The compositional structure of the supply chain within the PI milieu comprises indispensable entities, namely suppliers, PI centers, and retailers.
2. The geographical placements of the suppliers, PI-hubs, and retailers are pre-established, encompassing considerations of capacity constraints and transportation-related aspects, while the matter of siting-related predicaments remains extraneous to our analysis.
3. The intricacies of the replenishment process encompass vital dimensions, incorporating the influence of inventory holding costs, ordering costs, transportation costs, and the consequential ramifications of out-of-stock penalty costs.

### Model construction

#### Sets

- Suppliers (I): The set of all suppliers.
- PI-hubs (J): The set of all Physical Internet hubs.
- Retailers (K): The set of all retailers.

#### Parameter

- C_x: unit transport cost from supplier to PI-hub or between PI-hubs (yuan/(piece km))
- distance: the distance between nodes in the PI network (km)
- C_o: Ordering cost for replenishment from supplier or PI-hub (yuan/time)
- C_t: transport cost per unit distance (yuan/(piece.km))
- Demand_k: The demand of retailer k (pieces)
- C_s: retailer's unit inventory holding cost (yuan/day)
- Capacity_i: capacity of provider i (pieces)
- Capacity_j: Capacity of PI-hub j (pieces)

#### Variables

- x_ij: Quantity replenished from supplier i to PI-hub j
- y_jk: Quantity replenished from PI-hub j to retailer k
- z_i: Binary variable denoting source selection for replenishment of PI-hub j i stock
- w_jk: Binary variable representing the selection of sources from which PI-hub j replenishes retailer k's inventory

#### Replenishment model

The inventory replenishment problem in the PI environment can be properly transformed into a mixed integer programming problem. Here, we establish a formulation of the problem, describing the decision variables, objective function, and associated constraints, as follows:

Decision variables:

- x_ij: Quantity replenished from supplier i to PI-hub j
- y_jk: Quantity replenished from PI-hub j to retailer k
- z_i: Binary variable, indicating the choice of replenishment source for PI-hub i (0: means do not choose supplier i, 1: means choose supplier i)
- w_jk: A binary variable indicating purchases from PI-hub j to replenish retailer k's inventory (0: exclude PI-hub j as source, 1: include PI-hub j as source)
- D_kt: Represents the demand of retailer k at time t

Objective function:

The main objective is to minimize cumulative costs, including inventory holding costs, ordering costs, transportation costs, and penalty costs. The primary objective is to minimize cumulative costs, encompassing inventory holding, ordering, transportation, and penalty costs. The specific calculation formula of the total cost is shown in formula (1):\

1 $$\min \sum \begin{gathered} (i,j)[C\_x*dis\tan ce*x\_ij + C\_o*z\_i \hfill \\ + C\_t*(x\_ij + \sum {(k)y\_jk} ) + C\_s*(w\_jk*Demand\_k)] \hfill \\ \end{gathered}$$

2 $$\min \sum \begin{gathered} (i,j)[C\_x*dis\tan ce*x\_ij + C\_o*z\_i \hfill \\ + C\_t*x\_ij + C\_t*\sum {(k)y\_jk} + C\_s*w\_jk*D\_kt] \hfill \\ \end{gathered}$$

Restrictions:

1. Capacity constraints:
2. The quantity shipped from the supplier to the PI-hub should not surpass the supplier's designated capacity, and this constraint is expressed in the following Eq. (3):
  3 $$x\_ij \le Capacity\_i,\forall i,j$$
3. The volume of shipment from a PI-hub to a retailer must not exceed the capacity limit of the PI-hub, which is formally captured by the subsequent Eq. (4):
  4 $$y\_jk \le Capacity\_j,\forall j,k$$
4. Demand constraints:

The cumulative quantities supplied to the retailer from all PI-hubs should adequately fulfill the specific demand of the retailer, as rigorously manifested in the ensuing Eq. (5):

5 $$\sum {(j)y\_jk = Demand\_k,\forall k}$$

6 $$\sum {(j)y\_jk = D\_kt,\forall k} ,t$$

(3) Inventory balance constraints:
  The inventory magnitude at each PI-hub must appropriately satisfy the replenishment requisites of its affiliated retailers, as illustrated by the subsequent Eq. (7):
  7 $$\sum {(i)x\_ij - \sum {(k)y\_jk = 0,\forall j} }$$
(4) Source selection constraints:
(5) The binary variable z_i signifies the choice of source for replenishing the stock at PI-hub j, and its representation is given by the ensuing Eq. (8):
  8 $$z\_i \in \{ 0,1\} ,\forall i,j$$
  9 $$z\_i \in \{ 0,1\} ,\forall i$$
(6) The binary variable w_jk signifies the selection of sources to replenish the inventory of retailer k from PI-hub j, and its expression is articulated in Eq. (10):
  10 $$w\_jk \in \{ 0,1\} ,\forall j,k$$
(7) Solely one source, either supplier or PI-hub, can be chosen to replenish the inventory at PI-hub j, as concisely depicted in Eq. (11):
  11 $$\sum {(i)z\_i = 1,\forall j}$$
(8) Merely one source (PI-hub or retailer) can be chosen to replenish the inventory of retailer k from PI-hub j, as succinctly presented in Eq. (12):
  12 $$\sum {(i)z\_i*w\_jk} = 0,\forall j,k$$
  13 $$\sum {(i)z\_i + \sum {(j)w\_jk} } = 1,\forall k$$
(9) Demand constraint

The demand constraint ensure that the model can adapt to changing demand patterns over time.

14 $$\sum\nolimits_{j} {y_{\_jk} \ge D_{\_kt} } ,\quad \forall k,t$$

This constraint ensures that the total quantity supplied to retailer k from all PI-hubs at any given time t is at least equal to the demand D_kt.

## Algorithm design

### Simulated annealing algorithm (SA)

**Figure 2:**
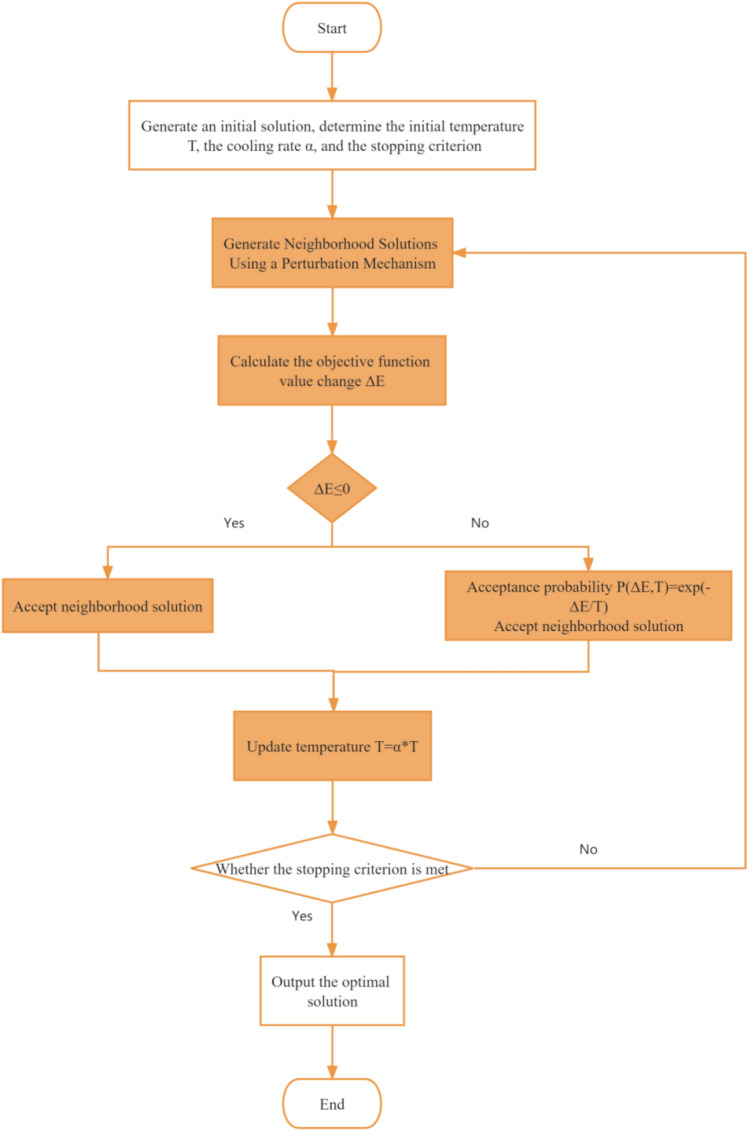
SA algorithm flow chart.

The Simulated Annealing Algorithm (SA) serves as a global optimization methodology, drawing inspiration from the annealing process observed in metallurgy. As a metaphorical mimicry of cooling material to attain a state of minimum energy, this algorithm endeavors to secure the best possible solution to a given problem through a progressive refinement of a candidate solution from a pool of potential solutions. Figure 2 illustrates the flow chart of the SA algorithm.

The underlying principle of this algorithm lies in its iterative nature, where it initiates from an initial solution and progressively improves it by generating a sequence of neighboring solutions. During each iteration, the algorithm judiciously assesses the objective function of both the current solution and its neighboring counterparts. Subsequently, the algorithm deliberates on accepting or rejecting these neighboring solutions by a probability distribution, influenced by the prevailing temperature. Over the course of its execution, the temperature gradually diminishes, affording the algorithm the capacity to overcome local minima and efficiently explore the expansive search space.

Consonant with the principle of simulated annealing, wherein cooling facilitates minimizing material energy, the objective function in the optimization context equates to the role of energy. By repeatedly traversing through a sequence of candidate solutions, the algorithm endeavors to discern the state of minimum energy, effectively resolving the optimization problem at hand.

The algorithm's calculation process unfolds as follows:

Step 1—Initialization: Commence by selecting an initial solution, alongside setting parameters, including the initial temperature T, cooling rate α, and a specified stopping criterion. The initial solution may be derived through random generation or heuristic methods. Temperature T and cooling rate α serve as pivotal controls governing the extent of search space exploration.

Step 2—Iteration: Embark on the iterative process, encompassing the following steps (a-e), until the stipulated stopping criterion is satisfied:

- Employ the perturbation mechanism to generate a neighboring solution. These neighboring solutions emerge by minutely altering the current solution, with the perturbation mechanism tailored to the specifics of the problem at hand.
- Calculate the variation in the objective function's value, denoted as *ΔE*, representing the disparity between the objective function value of the new solution and that of the current solution.
- If *ΔE* is negative (i.e., the new solution exhibits improvement), unequivocally accept the new solution as the incumbent one for the subsequent iteration.
- Conversely, if *ΔE* is positive (i.e., the new solution proves to be suboptimal), acceptance of the new solution transpires probabilistically based on the function *P(ΔE, T)* = *exp(-ΔE/T)*. This implies that, contingent upon the existing temperature and the degree of change in the objective function's value, a new solution may be embraced, even if it falls short of the current solution.
- Progressively update the temperature through the cooling schedule: *T* = α * T. This serves to lower the temperature, facilitating the forthcoming iteration while orchestrating the exploration of the search space.

Step 3—Stopping Criterion: The algorithm terminates upon meeting the designated stopping criterion, which may be contingent upon factors such as the number of iterations, the temperature reaching a predefined threshold, or other specified criteria.

The simulated annealing algorithm demonstrates prowess in resolving intricate combinatorial optimization problems, distinguished by their vast search space and the existence of numerous local minima. The algorithm's ability to transcend local minima by entertaining suboptimal solutions, as governed by the current temperature, combined with the gradual cooling of temperature, fosters a more efficient and expansive exploration of the search space.

### Genetic algorithm (GA)

**Figure 3:**
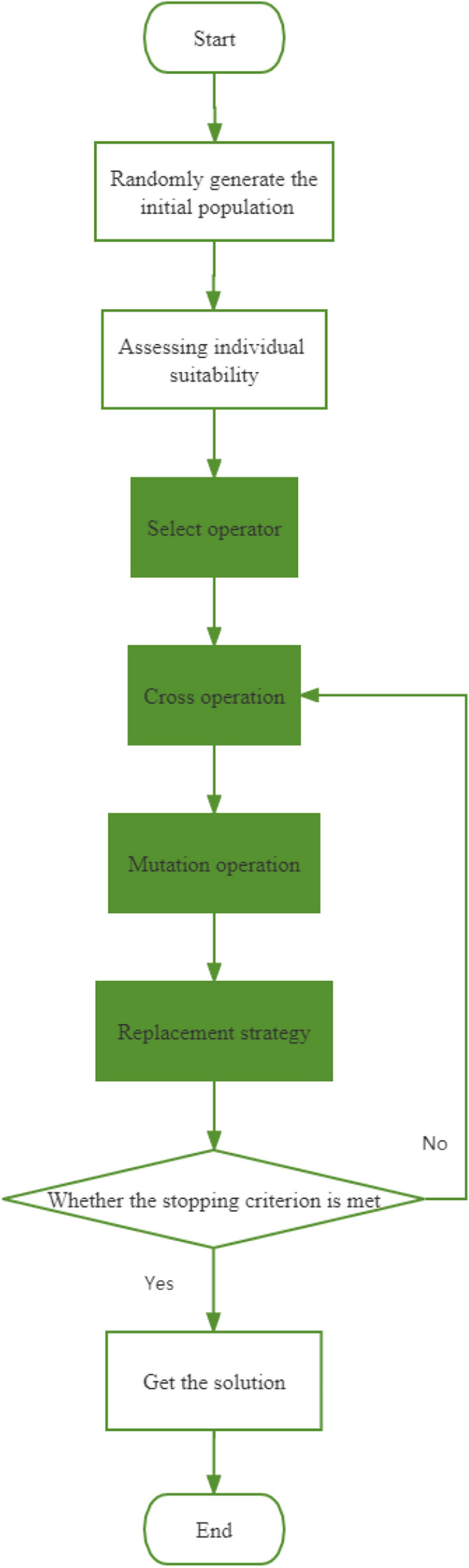
GA algorithm flow chart.

The Genetic Algorithm (GA) belongs to the class of optimization algorithms, drawing inspiration from natural selection and genetic processes. The algorithm operates upon a population of candidate solutions, symbolized as chromosomes, and progressively evolves by employing selection, crossover, and mutation operators. The flowchart illustrating the GA algorithm is presented in Fig. 3.

Step 1: At the outset, the GA algorithm generates an initial population of candidate solutions, which may be derived either randomly or through heuristic techniques. Each candidate solution finds representation as a chromosome, typically manifesting as a string of binary numbers. The population size is purposefully set to be sufficiently ample, affording ample diversity within the search space.

Step 2: The suitableness of each chromosome within the population is assessed by computing the value of the objective function for the corresponding solution. The fitness function, inherently contingent upon the specific problem, serves to gauge how effectively the solution addresses the optimization challenge.

Step 3: The selection operator comes into play, acting to elect parent chromosomes for reproduction, predicated on their respective fitness levels. Common selection methods encompass roulette selection and tournament selection. In roulette selection, each chromosome is assigned a probability of selection proportionate to its fitness. Alternatively, in tournament selection, a subset of chromosomes is randomly selected from the population, and the most suitable chromosome emerges as the designated parent.

Step 4: Crossover, signifying the process of amalgamating genetic material from two parental chromosomes to engender offspring, is executed. The crossover operation entails selecting a junction point on a chromosome and orchestrating the exchange of genetic material between the two parents. As a result, two new offspring chromosomes materialize, inheriting genetic information from both parents.

Step 5: The mutation operator is introduced to introduce random changes to the offspring's chromosomes. To prevent excessive perturbation to the genetic material, mutations are typically applied with low probability. Mutation involves random alterations to a few genes within a chromosome.

Step 6: The replacement operator takes charge of substituting the current population with the newly generated offspring. The replacement strategy may adopt a generational approach, wherein the entire population is supplanted by offspring, or a steady-state approach, wherein only a segment of the population undergoes replacement.

The algorithm perpetuates its iterative cycle, repeatedly traversing through steps 2 to 6, until a stipulated stopping criterion is met, such as reaching the maximum number of generations or attaining the targeted fitness level. The algorithm continues its iterative cycle, cycling through steps 2 to 6, until it meets a predetermined stopping criterion, such as reaching the maximum number of generations or achieving the desired fitness level. Ultimately, the final solution materializes as the chromosome manifests the highest fitness within the conclusive population.

GA algorithms effectively operate on a population of candidate solutions and leverage the selection, crossover, and mutation operators to engender progressively enhanced solutions. Through the iterative process encompassing population generation, fitness evaluation, parent selection, offspring generation, random alterations, and population replacement, the algorithm seeks to identify the optimal solution until the specified stopping criterion is fulfilled.

### Hybrid simulated annealing and genetic algorithm (SA–GA)

The Hybrid Simulated Annealing and Genetic Algorithm (SA–GA) entails the strategic amalgamation of the Genetic Algorithm (GA) and the Simulated Annealing Algorithm (SA). Within this innovative framework, GA is harnessed to optimize the SA, effectively capitalizing on the strengths of each method. The GA serves as a global exploration tool, adept at navigating the search space on a broader scale, while SA assumes the role of a local search method, skillfully annealing the offspring generated by GA. In essence, GA is entrusted with the task of generating offspring through crossover and mutation, while SA takes the reins in further optimizing these offspring before they are considered for selection and eventual inclusion in the succeeding generation.

**Figure 4:**
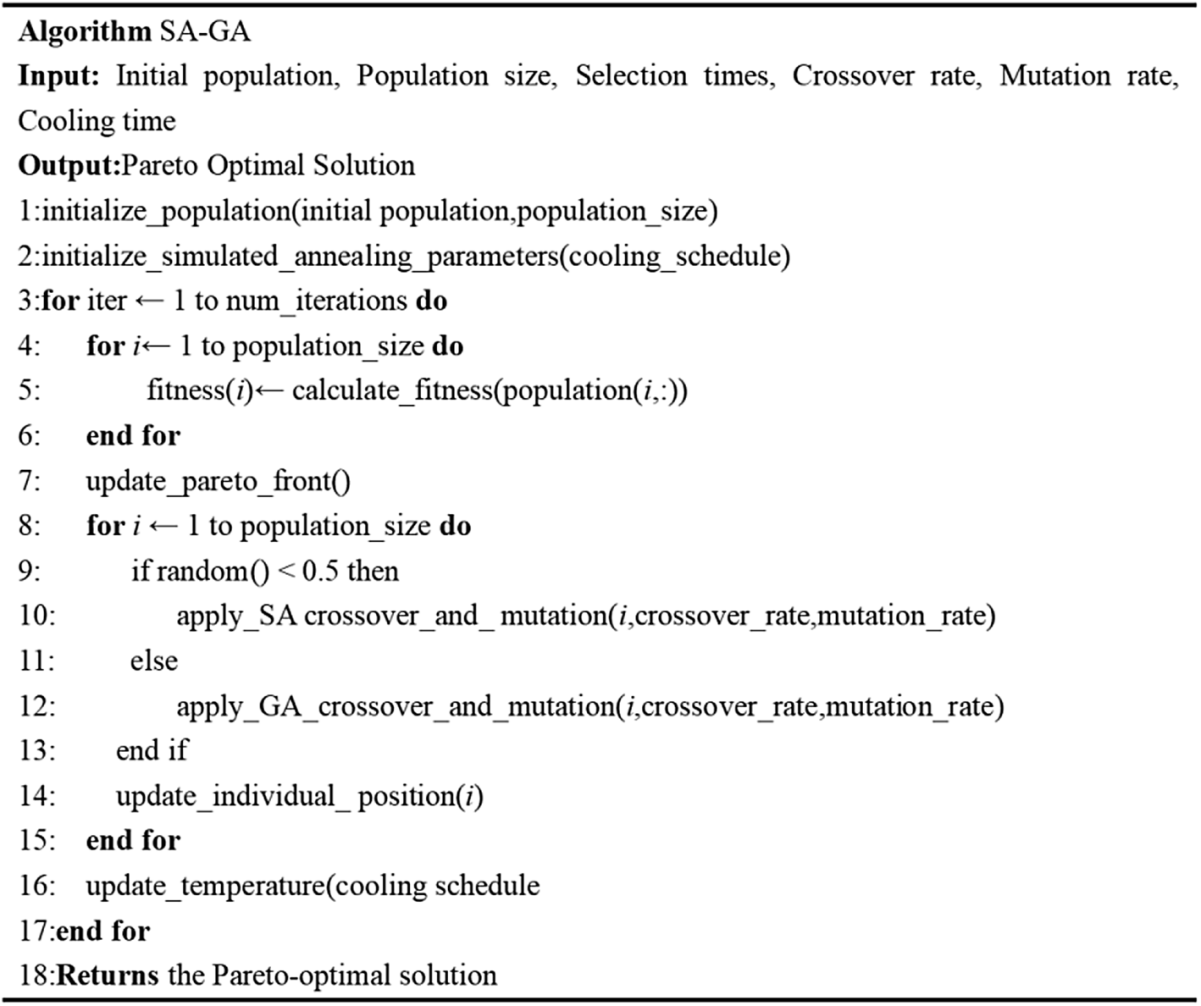
SA–GA algorithm pseudo code.

The overarching objective of SA–GA centers on elevating the algorithm's overall performance through the fusion of both methodologies. GA adroitly embarks on exploring the expansive search space, upholding a diverse cohort of candidate solutions, whereas SA excels in fine-tuning solutions and adroitly circumventing local optima. By harmonizing these two approaches, SA–GA endeavors to converge more effectively toward the true Pareto-optimal front, simultaneously preserving a diverse ensemble of solutions. The pseudocode illustrating the SA–GA methodology is aptly depicted in Fig. 4, and the specific steps of the algorithm are elucidated as follows:

Step 1: Initialization.

Generate an initial population of candidate solutions using either random or heuristic methods. Each solution comprises a combination of decision variables, encompassing the replenishment quantity from the supplier to the PI-hub (x_ij), the replenishment quantity from the PI-hub to the retailer (y_jk), and the source selection decisions for replenishment (z_i and w_jk).

Set the initial temperature and cooling schedule parameters, critical components within the Simulated Annealing (SA) phase.

Define the Genetic Algorithm (GA) parameters, including the population size, crossover and mutation rates, and termination criteria.

Step 2: Simulated Annealing (SA) Phase.

Commence with an initial solution for the Virtual Workbench (VW).

Proceed with a predefined number of SA iterations.

Execute a local search within the vicinity of the current solution by perturbing the decision variables. This entails adjustments to the replenishment quantities from the supplier to the PI-center (x_ij) or from the PI-center to the retailer (y_jk), and/or modifications to the source selection decisions (z_i and w_jk).

Evaluate the objective function for the new solution, incorporating the total cost to be minimized. The objective function accounts for inventory holding cost, ordering cost, transportation cost, and penalty cost, and is formulated as follows:

$$\begin{aligned} & {\text{Minimize }}\Sigma \left( {\text{i},\text{j}} \right) \, [\text{C}\_{\text{x}} \, *{\text{ distance }}* \, \text{x}\_{\text{ij}}\, + \,\text{C}\_{\text{o}} \, * \, \text{z}\_{\text{i}} \\ & \quad + \text{C}\_{\text{t}} \, * \, \left( \text{x}\_{\text{ij}}\, + \,\Sigma \left( \text{k} \right) \, \text{y}\_{\text{jk}} \right)\, + \,\text{C}\_{\text{s}} \, * \, \left( \text{w}\_{\text{jk}} \, *{\text{ Demand}}\_{\text{k}} \right)]. \\ \end{aligned}$$

In this context, C_x represents the unit transportation cost from the supplier to the PI-hub or between PI-hubs, distance denotes the distance between each node in the PI network, C_o signifies the ordering cost for replenishment from the supplier or PI-hub, C_t reflects the transportation cost per unit distance, C_s denotes the unit inventory holding cost of the retailer, and Demand_k signifies the demand of retailer k.

Accept or reject new solutions based on a probability function contingent upon the target value and the current temperature. The probability of acceptance is governed by the Metropolis criterion, conventionally employed within the SA algorithm.

Update the temperature by the cooling schedule, progressively diminishing the likelihood of accepting suboptimal solutions.

Reiterate the SA iterations until the temperature reaches the specified stopping criterion.

Step 3: Genetic Algorithm (GA) Phase.

Evaluate the fitness of each solution within the current population by computing the objective function value.

Employ selection operators (e.g., tournament selection or roulette selection) to elect parents for replication. The selection probability of each solution is contingent upon its fitness value.

Conduct crossover and mutation operations to engender offspring solutions. Crossover engenders new solutions by exchanging genetic information between the selected parent solutions, while mutation introduces small random changes to the solutions.

Assess the fitness of the offspring solution utilizing the objective function.

Implement elitism, integrating the best-performing solutions from the current population into the next generation to preserve their excellence.

Replace the current population with the descendant population.

Continue the genetic algorithm operation until a termination condition is met, such as reaching the maximum number of generations or achieving convergence.

Step 4: Termination.

Examine the termination conditions of the SA–GA algorithm, considering factors such as attaining the maximum number of iterations or achieving convergence of the solution. This determination can be predicated on the rate of improvement or stability of the objective function value.

If the termination condition is met, proceed to the next step. Otherwise, return to Step 2.

Step 5: Solution Extraction.

Extract the best solution from the final population, epitomizing the optimization of the replenishment process within a PI environment, to minimize the total cost while satisfying the retailer's inventory requisites.

Throughout the SA–GA algorithm, the decision variables x_ij, y_jk, z_i, and w_jk undergo manipulation and updating at each iteration to navigate the solution space. The objective function is rigorously evaluated to direct the search for an enhanced solution, pursuing the minimization of the total cost while simultaneously fulfilling the retailer's inventory requirements.

## Case verification

### Algorithm performance analysis

To evaluate the optimization effect of the SA–GA algorithm, four different benchmark functions (Sphere function, Rastrigin function, Ackley function, Griewank function) were selected for testing to compare the performance of SA–GA and SA individually. It is worth noting that both algorithms are executed on the PYTHON platform, the population size is set to 30, the maximum number of iterations is capped at 100, the problem dimension is set to 30, and the search space constraint of each dimension is set to [− 100, 100]. Each algorithm was subjected to 30 independent runs, which facilitated a comprehensive evaluation of its performance. Subsequently, the mean best fit, the standard deviation of the best fit, and the success rate (defined as the number of times the algorithm achieves the desired accuracy) are carefully recorded for analysis. The relevant results are shown in Table 1.

**Table 1:** Comparison table of SA–GA and SA algorithm test results.

Function	Algorithm	Average best fitness	Standard deviation	Success rate	Best resolution time (s)	Average resolution time (s)
Sphere	SA	1.55e-02	3.88e-03	0.67	0.0017	0.0021
	SA–GA	7.12e-04	1.62e-04	1	0.0921	0.1040
Rastrigin	SA	2.89	0.48	0.5	0.0025	0.0033
	SA–GA	1.92	0.32	1	0.1267	0.1769
Ackley	SA	2.15	0.19	0.53	0.0037	0.0046
	SA–GA	1.64	0.14	1	0.1814	0.2128
Griewank	SA	0.27	0.05	0.4	0.0031	0.0038
	SA–GA	0.16	0.04	1	0.1617	0.1825

Through careful analysis of the comparison results, it is evident that the SA–GA algorithm generally exhibits superior performance in terms of the average best fitness, standard deviation, and success rate compared to the SA algorithm among different test function choices.

### Case background introduction

This research initiative revolves around a specific equipment sales company, serving as the focal point of investigation. The company's operational workflow entails the parent company procuring equipment, which is subsequently distributed to each branch. Subsequently, the respective branches dispatch the equipment to corresponding distributors. In the context of this study, the parent company assumes the role of a supplier, while each branch corresponds to a designated PI hub. Furthermore, the distributors function analogously to retailers.

Of particular significance are the unit inventory holding costs at the PI-hub and retailer, which amount to 300 yuan/day and 500 yuan/day, respectively. Additionally, given that long-distance transportation costs are comparatively lower than last-mile transportation costs, the following assumptions are made: transportation cost from the factory to the PI-hub is 2 yuan/(piece km), transportation cost between PI-hubs stands at 2 yuan/(piece-km), and the transportation cost from the PI-hub to the retailer is quantified at 1.5 yuan/(piece-km). Moreover, the order cost for PI-hubs to procure from suppliers entails an amount of 1000 yuan per transaction, similarly, orders placed between PI-hubs incur a cost of 1000 yuan per transaction, and retailers placing orders with PI-hubs are subjected to a charge of 1000 yuan per transaction. Importantly, the penalty cost is determined to be 30% of the value of the goods at the retailer.

Accounting for the relatively substantial distance between the supplier and the PI-hub, the order lead time for the PI-hub to procure from the supplier is set at 38 days. Similarly, the order lead time for transactions between PI-hubs spans 20 days, while the order lead time for retailers to procure from the PI-hub encompasses 15 days. Tables 2, 3, 4, 5 and 6 further enlists essential location information and demand particulars, critical to the context of this study.

**Table 2:** Distance between suppliers and PI-hub (unit: km).

Supplier code	Pi-hub (1)	Pi-hub (2)	Pi-hub (3)	Pi-hub (4)
1	3172	3025	3332	2559
2	3271	3302	3512	2438

**Table 3:** Distance between PI-hub and PI-hub (unit: km).

PI-hub number	Pi-hub (1)	Pi-hub (2)	Pi-hub (3)	Pi-hub (4)
Pi-hub (1)	–	1137	1224	1578
Pi-hub (2)	1137	–	1316	1645
Pi-hub (3)	1224	1316	–	1221
Pi-hub (4)	1513	1645	1221	–

**Table 4:** Distance between PI-hub and retailer (unit: km).

Retailer number	Pi-hub (1)	Pi-hub (2)	Pi-hub (3)	Pi-hub (4)
1	540	764	768	785
2	463	774	715	327
3	308	469	482	291
4	485	636	1043	288
5	587	777	1060	332
6	287	737	1315	587
7	155	630	1402	666
8	352	820	1029	658

**Table 5:** Retailers' average demand for PI-hubs (unit: pieces).

PI-hub number	Average	Variance
1	59	7
2	51	5
3	54	6
4	52	6

**Table 6:** Customers' average demand for retailers (unit: pieces).

Retailer number	Average	Variance
1	52	5
2	52	7
3	61	9
4	60	8
5	52	8
6	55	7
7	53	5
8	52	5

### Analysis of case results

**Figure 5:**
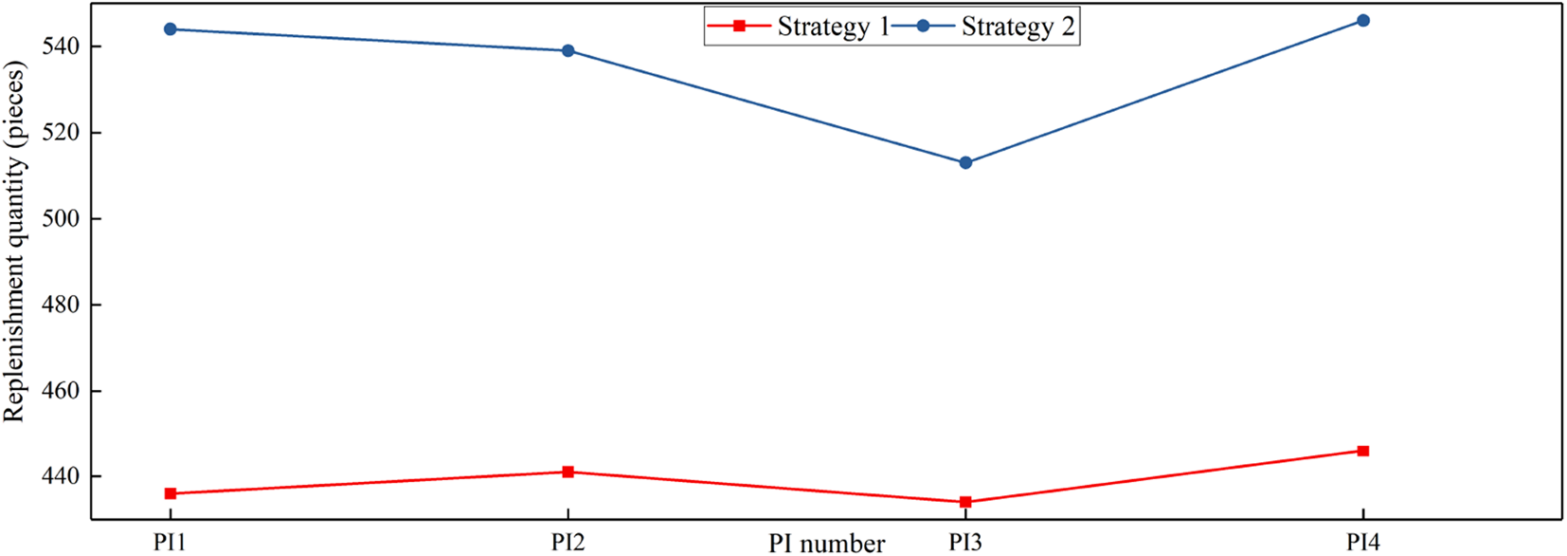
Replenishment demand of different PI hubs under strategy 1 and strategy 2.

**Figure 6:**
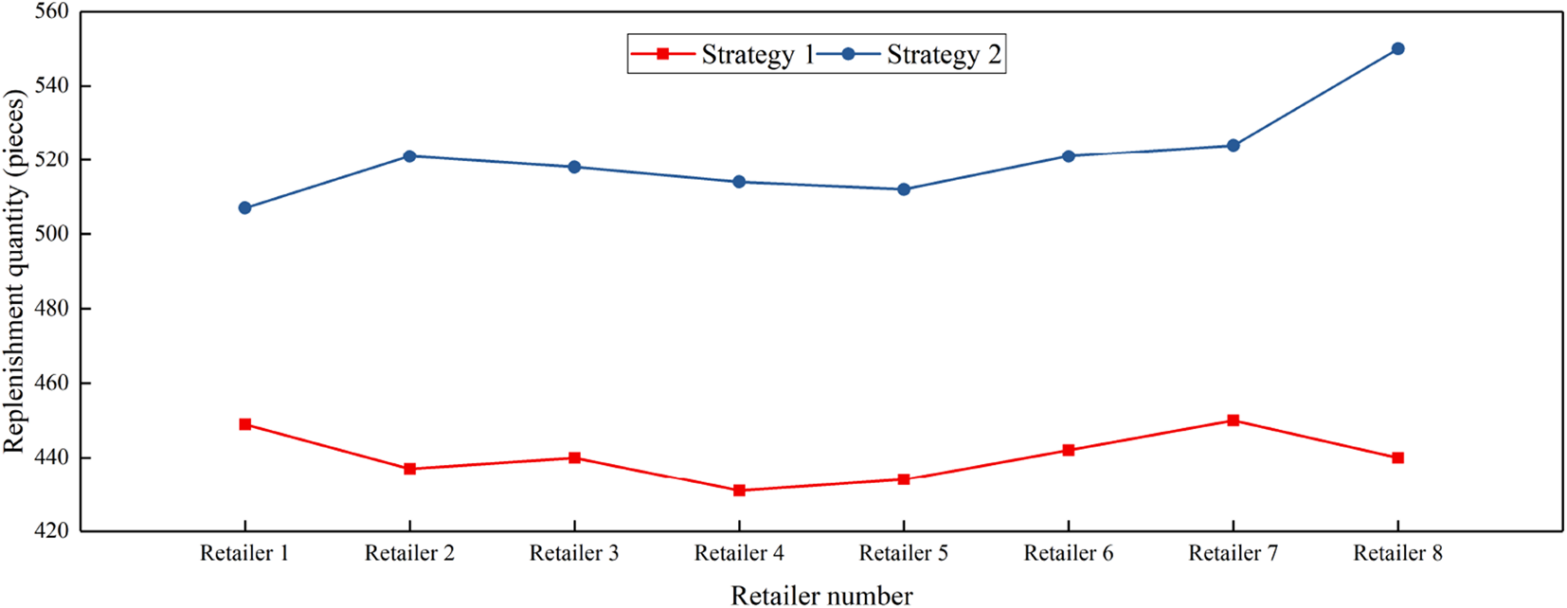
Replenishment requirements of different retailers under strategy 1 and strategy 2.

The outcomes of the case study are presented in Figs. 5 and 6, depicting the number of replenishment pieces in different PI hubs and each retail outlet, respectively, under the two distinct replenishment strategies. A meticulous examination of the results in both figures yields noteworthy insights. Specifically, it becomes evident that the number of replenishment items in the PI hub, under replenishment strategy 1, is notably lower than that observed under strategy 2. This observation signifies that strategy 1 effectively capitalizes on the interconnected nature of the PI network, facilitating a seamless flow of goods between PI hubs and retailers. By leveraging the network's collaborative potential, strategy 1 adeptly optimizes the transfer efficiency of goods sources, effectively meeting the actual demand with greater efficacy.

In stark contrast, strategy 2 falls short in harnessing the interconnectivity aspects of the PI network. As a result, it necessitates replenishment through suppliers, thereby leading to a comparatively reduced cargo transfer efficiency when compared to strategy 1. This discrepancy highlights the critical advantage of strategy 1 in attaining higher efficiency and responsiveness in meeting demand while efficiently managing the flow of goods within the interconnected PI network. The contrasting outcomes underscore the significance of embracing the network's collaborative potential, thereby enhancing overall supply chain efficiency and resilience in the context of the equipment sales company.The core advantage of Strategy 1 lies in its strategic alignment with the interconnected nature of the PI network. It isn’t merely a replenishment strategy but a harmonious integration into the PI ecosystem, ensuring that the flow of goods between PI hubs and retailers is not just maintained but optimized. Every piece of data points towards a reduction in total costs and penalty amounts, painting a narrative of efficiency and cost-effectiveness that is absent in Strategy 2.

To facilitate a more lucid comparison of cost values between Strategy 1 and Strategy 2, we present a numerical representation of the funds expended during the cargo transportation process under each strategy in Table 7.

**Table 7:** Comparative analysis of each cost under the two source selection strategies (unit: yuan).

Cost type	Strategy 1	Strategy 2
PI total cost	112,563.33	154,453.94
Supplier ordering cost	0.55	0.92
PI ordering cost	50.18	34.97
Vendor-PI shipping costs	4079	7949
PI-PI shipping cost	28,342	32,479
PI holding cost	65,335.12	95,332.62
PI—retailer out-of-stock cost	25,126.30	62,531.91
Retailer total cost	59,863.33	83,014.15
Retailer order cost	89.72	99.89
PI—Retailer shipping cost	9543.55	14,860.38
Retailer carrying costs	48,842.57	69,982.11

Upon examination of the results in Table 7, it is evident that both the total cost of PI and the total retail cost incurred by Strategy 1 are significantly lower than those incurred by Strategy 2. This outcome further corroborates the efficacy of the optimization objective proposed in the replenishment model put forth in this study. Concurrently, the penalty amount associated with Strategy 1 is notably smaller than that incurred by Strategy 2, aligning with the findings depicted in Figs. 5 and 6. This contrast can be attributed to the proximity between PI hubs and retailers, as well as the considerable distance between suppliers and PI hubs.

Strategy 1 predominantly relies on the efficient flow of goods between PI hubs and suppliers to facilitate the replenishment process. Only when the inventory at a PI hub is insufficient would replenishment through suppliers be initiated, thereby ensuring swift and timely goods transfer, leading to lower penalty costs. In contrast, strategy 2 leans heavily on suppliers for replenishment, necessitating goods to traverse longer distances, resulting in lengthier replenishment processes and, consequently, higher penalty costs.

The effectiveness of the SAGA algorithm is further corroborated by the cost analysis depicted in Table 7. Strategy 1, underpinned by SAGA, demonstrates a significant reduction in both the total PI and retail costs, in addition to lower incurred penalty costs. These findings are indicative of the algorithm’s capability to optimize multiple cost factors concurrently, affirming its role in enhancing the economic efficiency of Strategy 1.

In the context of the PI network’s complexity, the SAGA algorithm facilitates an optimized flow of goods between PI hubs and retailers, minimizing dependency on suppliers for replenishment. This optimization is reflected in the reduced total and penalty costs associated with Strategy 1, affirming the algorithm’s effectiveness in real-world PI ecosystems.

In conclusion, the empirical data underscores Strategy 1’s efficiency and cost-effectiveness, attributes that are significantly enhanced by the SAGA algorithm. The algorithm’s role in transforming theoretical constructs into practical, implementable strategies is evident, marking a significant contribution to the field of PI inventory management.

### Ethical approval and consent to participate

This article does not involve any moral and ethical disputes, and this article does not involve any human-related experiments. All the authors will participate in the review and publication process.

## Discussion

### Innovation and effectiveness of the proposed strategy

The proposed inventory replenishment strategy is introduced in a context where existing literature and methodologies are limited, particularly within the Physical Internet (PI) framework. This study contributes a nuanced approach that is both responsive and adaptive to the dynamic nature of PI, addressing specific challenges associated with cost, efficiency, and demand fulfillment.

The strategy’s innovation lies in its integration of real-time data and dynamic decision-making processes, optimizing the movement of goods between PI centers and retailers. Empirical results indicate a marked reduction in associated costs and enhanced efficiency compared to traditional static replenishment methods (Strategy 2).

### SA–GA algorithm: a computational advancement

The introduction of the SA–GA algorithm is another pivotal aspect of this study. It is a hybrid model combining the strengths of Simulated Annealing (SA) and Genetic Algorithm (GA), designed to optimize complex, multi-dimensional problems inherent in PI inventory management.

The algorithm’s innovation is evidenced by its enhanced convergence speed and solution accuracy. It adeptly balances exploration and exploitation processes, mitigating the limitations of standalone SA, particularly its susceptibility to local optima and slow convergence in complex problem spaces.

### Comparative analysis

A detailed comparative analysis underscores the proposed strategy and SA–GA algorithm’s superiority. Given the limited directly comparable studies in the field, the analysis extends to traditional inventory management approaches and algorithms. Metrics of cost savings, computational efficiency, and solution accuracy serve as benchmarks, highlighting the significant advancements introduced by this study.

### Conclusion of discussion

This study’s contributions are anchored in the innovative strategy and SA–GA algorithm, offering tangible solutions to the intricate challenges of inventory management within the PI. The empirical results affirm the strategy’s applicability and the algorithm’s computational prowess, marking a significant step forward in the field.

The academic discourse on inventory management within the PI is enriched by these contributions, offering new avenues for research and practical applications. Future studies may build upon this foundational work, exploring further optimizations and adaptations to cater to the evolving landscape of the Physical Internet.

## Conclusion

This study addressed the complex issue of inventory replenishment in a Physical Internet (PI) environment. We formulated an optimization model aimed at minimizing inventory holding costs, ordering costs, shipping costs, and penalty costs, a challenge prevalent in contemporary supply chain management.

The introduction of the hybrid optimization algorithm SAGA (Simulated Annealing and Genetic Algorithm) marked a pivotal point in our research. A systematic comparison between the SA and SA–GA algorithms revealed the superior efficacy of the latter, as evidenced by enhanced performance metrics including average best fitness, standard deviation, and success rate.

A practical case study further substantiated our theoretical findings. We evaluated two distinct inventory replenishment strategies. Strategy 1, which integrated PI networks, demonstrated a notable reduction in the number of replenishment items and overall costs compared to Strategy 2, which was grounded in conventional inventory management practices. These empirical findings underscore the significant role of network connectivity in optimizing inventory management.

Quantitatively, Strategy 1 yielded a reduction of 41,890.61 yuan in total cost and 37,405.61 yuan in penalty costs compared to Strategy 2. These results not only validate the effectiveness of our proposed optimization model and hybrid algorithm but also highlight the potential impact of IoT technologies in enhancing supply chain efficiency.

In conclusion, the findings of this study offer valuable insights into the advancement of inventory management within the PI environment. The demonstrated efficacy of the SAGA algorithm and the cost-effectiveness of Strategy 1, grounded in interconnected PI networks, suggest promising avenues for future research and practical applications in supply chain management optimization. Our contributions lie in the successful amalgamation of theoretical constructs with empirical validations, paving the way for innovative, efficient, and cost-effective inventory management solutions in the realm of the Physical Internet.In addition to the theoretical contributions and empirical findings, this study offers several managerial insights. Firstly, the implementation of the SA–GA algorithm in inventory management within the Physical Internet environment demonstrates a significant reduction in costs and improvement in efficiency, suggesting that businesses should consider adopting such advanced optimization techniques. Secondly, the comparative analysis between traditional inventory management approaches and the proposed strategy underscores the importance of embracing innovative technologies and algorithms to stay competitive in the rapidly evolving supply chain landscape. Finally, our findings highlight the potential of interconnected PI networks in enhancing supply chain efficiency, suggesting that managers should explore the integration of IoT technologies to optimize their operations.

## Limitations and future work

### Limitations

Despite the encouraging findings and contributions of this paper, there are some limitations worth acknowledging:

1. Simplifying assumptions: The inventory replenishment model relies on certain assumptions, such as predetermined locations of suppliers, PI centers, and retailers, fixed transportation costs, and constant demand. While these assumptions aid in model development, they may not fully capture the complexities that exist in real-world supply chain scenarios.
2. Limited case study: The actual case study conducted in this paper is based on a specific equipment sales company. Although it provides valuable insight into the benefits of Strategy 1, the general applicability of the results to other industries or different supply chain environments remains to be explored.
3. The SA–GA algorithm has demonstrated significant efficacy in optimizing the proposed dynamic replenishment strategy. However, the study did not extend its comparative analysis to include other well-established algorithms in the field. Algorithms such as Particle Swarm Optimization (PSO), Ant Colony Optimization (ACO), and others renowned for their optimization capabilities were not evaluated alongside SA–GA.

### Future work

To address the identified limitations and advance knowledge in the field, the following avenues for future research are suggested:

1. Realistic Scenario Modeling: Future research can consider more realistic and dynamic scenarios, incorporating uncertainties such as demand fluctuations, changes in transportation costs, and dynamic locations of suppliers, hubs, and retailers. This will enhance the applicability of the model in the wider supply chain context.
2. Case studies in different industries: Conducting case studies across different industries and supply chain configurations will help to gain a more complete understanding of the applicability and effectiveness of the proposed inventory replenishment model. Comparing the results in different contexts will highlight the strengths and limitations of the method.
3. Future research endeavors could aim to bridge this gap by conducting an exhaustive comparative analysis, evaluating the SA–GA algorithm against other algorithms on parameters like computational efficiency, solution accuracy, and adaptability to dynamic PI environments. This would not only validate the findings of this study but also contribute to the ongoing discourse on optimizing inventory management within the PI.

In conclusion, while this paper presents an important step toward optimizing inventory replenishment in the context of the physical Internet, there are still areas for further exploration and refinement. By addressing limitations and pursuing future research directions, we can continue to advance the field of PI-based supply chain management and pave the way for more resilient, efficient, and sustainable supply chains in the future.

## Data Availability

Data will be provided on request.If anyone would like data from this study, please contact the corresponding author of this article.
